# Deciphering the impact of air frying vs. oven roasting on crayfish (*Procambarus clarkii*) quality: A multiscale journey from water migration and protein denaturation to texture, flavor, and digestibility

**DOI:** 10.1016/j.fochx.2026.103495

**Published:** 2026-01-05

**Authors:** Wensi Xu, Shengcai Xu, Aihua Deng, Xiaoyang Liu, Liang Song, Dayong Zhou, Qifu Yang

**Affiliations:** a College of Life and Environmental Sciences, Hunan University of Arts and Science, Changde 415000, PR China; b SKL of Marine Food Processing & Safety Control, Dalian 116034, PR China; c Hunan Provincial Engineering Research Center for Healthy Aquaculture and Processing of Shrimp and Crab, Changde 415000, PR China; d National Engineering Research Center of Seafood, Dalian 116034, PR China; e School of Food Science and Technology, Dalian Polytechnic University, Dalian 116034, PR China

**Keywords:** Crayfish, Dry-heat treatment, Water distribution, Microscopic architecture, Sensory attributes, Digestion characteristics

## Abstract

Crayfish are valued for their culinary appeal and nutritional profile. This study compares air frying (AF) and roasting (RO) impacts on crayfish muscle. We investigated water migration, protein structural changes, microstructure, sensory attributes, digestibility, and volatile compounds. Dry-heat processing induced significant protein oxidation and aggregation, as evidenced by a 3.6-fold increase in carbonyl content, a 48 % rise in the myofibrillar fragmentation index, and a reduction in free sulfhydryl groups. AF promoted protein oxidation due to rapid heating, whereas RO enhanced proteolysis. AF samples retained bound water within compact matrices, restricting water mobility, whereas RO formed porous networks that increased free water content, yielding smaller digesta particles (367.9 nm). AF enriched volatile compounds through dehydration-driven concentration, with tetrahydro-2-furanmethanol as a discriminant marker. AF at 170 °C/10 min achieved optimal sensory acceptance. Our findings elucidate multiscale mechanisms linking water-protein dynamics to texture, flavor, and digestibility, providing a scientific basis for optimizing crustacean processing.

## Highlights

• Dry-heat causes oxidative degradation, higher carbonyl and MFI, lower sulfhydryl.
• Air frying oxidizes proteins, roasting degrades them.
• Air frying retains bound water via compact matrices, concentrating volatiles.
• Roasting boosts digestibility with porous architectures via proteolysis.
• Tetrahydro-2-furanmethanol acts as an authentication marker for air-fried crayfish.

## 1 Introduction

Crayfish (*Procambarus clarkii*) is an important freshwater aquaculture resource prized for its taste and nutritional value, including a high protein content (Xu et al., 2024). Its production, especially in China, has seen substantial growth (National Fisheries Technology Extension Center & Chinese Society of Fisheries, 2024). Various thermal processing methods, such as steaming, boiling, and frying, are employed, each distinctly influencing the final flavor and quality of crayfish (Fan et al., 2020; Xu et al., 2024; Zhou et al., 2024). Among these, dry-heat treatments like air frying and roasting are significant for creating unique sensory attributes.

Air frying and roasting are two prevalent dry-heat methods that impart desirable textures and flavors to foods. However, they also induce significant moisture loss and protein denaturation, which can adversely affect physicochemical properties, sensory quality, and nutritional value (Wang et al., 2023). For instance, although air-fried crayfish may achieve high consumer acceptance, the process can promote protein aggregation that compromises digestibility (Zhou et al., 2022; Suleman et al., 2020; Bhat et al., 2021). Similarly, roasting often leads to excessive dryness, underscoring the need for better process control (Lian et al., 2023). Therefore, understanding the dynamic changes in water status and its interaction with proteins is fundamental to controlling the quality of cooked crayfish (Bi et al., 2023; Lian et al., 2023). However, the systematic mechanisms of how different dry-heat methods distinctly regulate water distribution and protein structure at multiple scales, and how these changes subsequently define multisensory quality and digestibility in crayfish, remain inadequately explored. Consequently, a comparative investigation into how air frying and roasting differentially govern quality formation through water-protein interactions constitutes a pivotal scientific question.

This study deciphers quality changes in crayfish (*Procambarus clarkii*) under dry-heat processing through a multiscale investigation. We systematically examine water migration dynamics, protein oxidative denaturation, microstructural evolution, sensory attributes, and digestibility patterns to compare mechanistic distinctions between air frying and oven roasting. The central objectives were to elucidate three interdependent phenomena: (1) the dynamic redistribution of water states (bound, immobilized, and free water) during thermal processing; (2) protein structural responses spanning oxidative degradation to conformational reorganization; and (3) the consequential relationships linking physicochemical modifications to sensory properties and in vitro digestibility. By integrating these analytical dimensions, this work establishes how processing modality governs crayfish quality architecture. Findings provide actionable insights for industrial crayfish processing parameter optimization, enhancing product excellence while advancing fundamental knowledge of aquatic resource thermal processing.

## 2 Materials and methods

### 2.1 Sample preparation

Fresh crayfish (*Procambarus clarkii*) with an average weight of 21.65 ± 1.83 g were purchased from a local RT-Mart supermarket in Changde, China. The crayfish were thoroughly rinsed under running tap water. To simulate common culinary practices in many Asian and European regions where crayfish are often cooked intact, whole crayfish were used for processing. Although the cephalothorax (containing the hepatopancreas) and intestine are often discussed in terms of potential contaminant accumulation, this study focused specifically on the physicochemical and sensory properties of the abdominal muscle (the main edible tail meat). To ensure microbial safety, a thorough heating protocol was implemented, requiring the core temperature to be maintained above 75 °C for 10 min. The crayfish were cooked using two methods: air frying (AF) at 170, 180, and 190 °C, and oven roasting (RO) at 200, 210, and 220 °C, all for a duration of 10 min. Each processing group consisted of 20 crayfish. The core temperature was monitored at 2-min intervals between the 4th and 10th minutes using an external dual-channel data logger (testo 175 T3, Testo Instruments International Trade Co., Ltd., Shanghai, China) equipped with separate probes inserted into the cephalothorax and abdomen.

### 2.2 Moisture content measurement

The moisture content analysis of crayfish tail meat was conducted in accordance with the GB 5009.3–2016 direct drying protocol. Moisture content was determined using a DSH-50-5 rapid moisture meter (Yueping Scientific Instrument, Shanghai).

### 2.3 Low-field nuclear magnetic resonance (LF-NMR) and magnetic resonance imaging (MRI) analysis

Crayfish tail meat samples (3.0 ± 0.5 g) were analyzed using LF-NMR and MRI with a Niumag NMI20-030H-1 nuclear magnetic resonance analyzer (Suzhou, China). All parameters were kept consistent with the findings reported by Liang et al. (2022), except for the TM value (set to 3000.00 ms) and NECH (set to 3000).

### 2.4 Protein oxidation determination

The determination of protein carbonyl level in crayfish tail meat was conducted using a commercially available protein carbonyl content assay kit (Acmec Biochemical Technology Co., Ltd., Shanghai, China).

The quantification of free sulfhydryl group content adhered to the methodology outlined by Xu et al. (2024). Briefly, 1.5 mL crayfish protein solution (5 mg/mL) was blended with 10 mL Tris-glycine buffer (4 mM EDTA, 90 mM glycine, 86 mM Tris, 0.5 mL of 10 mM DTNB, pH 8.0). After 1 h incubation at 25 °C, samples were centrifuged (4 °C, 10 min) and free sulfhydryl groups quantified from supernatant absorbance at 412 nm.

### 2.5 Protein degradation determination

The TCA-soluble peptide content in crayfish tail meat was quantified following the method by Xu et al. (2025). A 3 g sample of crayfish meat was mixed with 27 mL of a 5 % trichloroacetic acid (TCA) solution, homogenized at 1000 rpm for 1 min, and then centrifuged at 5000 ×*g* for 10 min. The supernatant was collected, diluted 25-fold, and its absorbance at 562 nm was measured using the BCA method to quantify the soluble peptide content.

The myofibril fragmentation index (MFI) of crayfish tail meat was determined according to the method described by Xu et al. (2025). Crayfish meat sample (2 g) was combined with 40 mL of chilled buffer (pH 7.0) and centrifuged at 4000 ×*g* for 10 min. The resulting pellet was washed three times. The final precipitate was resuspended in 10 mL of buffer, and its absorbance was measured at 540 nm. The MFI was calculated by multiplying the absorbance value by 200.

### 2.6 Measurement of surface hydrophobicity

Surface hydrophobicity of crayfish tail meat protein was analyzed according to the method described by Xu et al. (2024). This involved examining a protein solution (2 mg/mL) with bromophenol blue (BPB) at 1 mg/mL. A phosphate buffer without protein served as a control. After shaking at room temperature for a set period, all samples and the control were centrifuged at 2000 ×*g* for 15 min at 4 °C. The supernatant was then diluted tenfold with PBS, and the absorbance at 595 nm was measured.

### 2.7 Intrinsic fluorescence emission analysis

Intrinsic fluorescence emission was analyzed as previously described (Xu et al., 2024). The intrinsic fluorescence emission of a 1 mg/mL crayfish tail meat protein solution was systematically analyzed using a fluorescence spectrophotometer from Hitachi Corporation (Tokyo, Japan). The excitation wavelength was set at 280 nm, and the emission spectrum was recorded from 290 to 450 nm.

### 2.8 Fourier transform infrared (FTIR) spectroscopy analysis

Protein secondary structures were identified using FTIR spectroscopy, following the method outlined by Xu et al. (2024). Crayfish tail meat protein samples were combined with dried KBr powder and pressed into thin pellets. FTIR spectra were recorded using a Perkin Elmer spectrometer (Salem, MA) over the wavenumber range of 400–4000 cm^−1^. The 1600–1700 cm^−1^ region was analyzed to identify protein secondary structures using PeakFit v4.12 software (SeaSolve Software Inc., USA).

### 2.9 Microstructure analysis

Microstructure analysis of crayfish tail meat transverse sections followed Xu et al. (2024) methodology, samples fixed in 10 % formaldehyde (24 h), paraffin-embedded, sectioned, H&*E*-stained, and examined via bright-field microscopy (Nikon ECLIPSE Ti—S inverted microscope).

Following the method of Xu et al. (2025), the microstructure of crayfish tail meat was analyzed at 4000× magnification using a cold-field emission SEM (SU8010, HITACHI, Tokyo, Japan). The samples were rapidly frozen in liquid nitrogen, transferred to a vacuum chamber, and freeze-dried at −90 °C for 30 min. After drying, they were gold-coated and observed under the SEM at 2 kV accelerating voltage.

### 2.10 Texture profile analysis

A TA. XT Plus texture analyzer (Stable Micro Systems LTD, Godalming, UK) quantified textural parameters (hardness, springiness, chewiness, resilience) in the second abdominal segment of crayfish tails. The evaluation was performed under ambient conditions at 25 °C, following the methodology described by Xu et al. (2024). The testing parameters included a trigger force of 5 g, pretest speed of 1.0 mm/s, test speed of 3 mm/s, post-test speed of 5 mm/s, compressed depth of 25 %, time interval of 5 s, and a compression ratio set at 50 %.

### 2.11 Color profile analysis

The color of dorsal and ventral regions of crayfish tail meat was analyzed at 25 °C using a ColorFlex EZ 45/0 instrument (HunterLab, VA, USA). Measured parameters comprised L* (brightness), a* (redness/greenness), and b* (yellowness/blueness). Hue-angle (H^o^) and ΔE were derived from equations by Liang et al. (2022) and Wang et al. (2024).

### 2.12 Cooking loss measurement

Whole crayfish samples were cut into uniform pieces and weighed. After dry-heat cooking, samples were cooled, and reweighed. Cooking loss was calculated as a percentage using the formula described by Wang et al. (2023).

### 2.13 Water holding capacity (WHC) determination

The WHC of crayfish tail meat was determined by following the method described by Li et al. (2022). Crayfish samples (2 *g*) were centrifuged (10,000 ×*g*, 15 min, 4 °C). The weight was measured before (M_0_) and after (M_1_) centrifugation. WHC was calculated as the percentage of M_1_ relative to M_0_.

### 2.14 Sensory evaluation

Sensory evaluation was performed by a trained panel (*n* = 10) from the Hunan University of Arts and Science. Cooked crayfish samples (air-fried or roasted) were assessed for appearance, aroma, texture, taste, and overall acceptability. The sensory evaluation conducted was exempt from formal ethical review approval in accordance with the policies of Hunan University of Arts and Science and the national regulations for minimal-risk food studies involving adult volunteers. All participants were provided with written and oral information regarding the study's purpose, procedures, and their rights, including the anonymity of their responses and the freedom to withdraw at any time without consequence. Written informed consent was obtained voluntarily from each participant prior to the evaluation sessions. All collected data were anonymized and handled confidentially.

### 2.15 Analysis of gastrointestinal digestion in vitro

#### 2.15.1 Static in vitro gastrointestinal digestion protocol

The specimen was digested in vitro using a gastrointestinal simulation method from Wang et al. (2023). Samples (frozen-dried crayfish tail meat 100 mg) were mixed with 10 mL of simulated salivary fluid and incubated at 37 °C for 10 min. Then, 10 mL of simulated gastric fluid (containing 1.2 g of pepsin with an activity level of 400 U/mg) was added, and the mixture was incubated at 37 °C for 2 h. After gastric digestion, the pH was adjusted to 7.0, and simulated intestinal fluid (containing 96 mg of trypsin with an activity level of 250 U/mg) was added. This was followed by another 2-h incubation at 37 °C. The digestion process ended by boiling the mixture for 1 min to stop enzyme activity. The supernatant was collected after centrifuging at 10,000 ×g for 10 min and used for further analysis.

#### 2.15.2 Digestive properties measurement

The determination of the insoluble residues ratio was conducted using the method outlined by Wang et al. (2024). Centrifugation of gastric and intestinal digests (12,000 ×g, 10 min, 4 °C) yielded pelleted insoluble residues. After supernatant removal and drying to constant mass, their proportional content was quantified.

The soluble amino group content was quantified using o-phthalaldehyde (OPA) with amino groups, and a standard curve was created using l-serine as the reference (Duque-Estrada et al., 2019).

The soluble peptide content was measured using the method outlined by Wang et al. (2023). High-molecular-weight proteins were precipitated with trifluoroacetic acid (TCA), and the peptide concentration in the supernatant was determined using the Lowry method, with bovine serum albumin (BSA) as the reference standard.

#### 2.15.3 Post-digestion particulate profiling

The gastric and intestinal digesta supernatants were subjected to particle size distribution analysis using a laser diffraction analyzer (Malvern 3000 HSA, UK) (Zhang et al., 2024).

### 2.16 Volatile compounds analysis

Volatile compounds analysis followed Ma et al. (2020) using GC–MS with SPME autosampler. Tail meat volatile compounds were extracted by 50/30 μm DVB/CAR/PDMS fiber (Anpel, China), cyclohexane as an internal standard. The sample was equilibrated at 80 °C for 30 min, followed by headspace extraction under the same temperature for 30 min. The fiber was then desorbed in the GC injection port for 5 min. Analysis was conducted on an Agilent 7820 A GC-5977E MSD system equipped with an HP-5MS capillary column (30 m × 0.25 mm, 0.25 μm). The temperature was programmed as follows: hold at 35 °C for 3 min, ramp at 4 °C/min to 200 °C, then at 20 °C/min to 260 °C and hold for 8 min. Helium carrier gas was used at a flow rate of 1.5 mL/min in splitless mode. The MS conditions included electron impact ionization at 70 eV, an interface temperature of 280 °C, and a mass scan range of 30–500 *m*/*z*. Compounds were identified via NIST 23. L mass spectral library (match threshold >80 %).

### 2.17 Statistical analysis

Triplicate data expressed as mean ± SD. Significance (*p* < 0.05) assessed by one-way ANOVA (SPSS 16.0). Data visualization and further statistical analysis were performed using Origin Zhang et al., 2025 (OriginLab Corporation, Northampton, MA, USA). Additionally, partial least squares-discriminant analysis (PLS-DA) was carried out with MetaboAnalyst 6.0 (https://www.metaboanalyst.ca), including a permutation test and calculation of variable importance in projection (VIP) scores.

## 3 Results and discussion

### 3.1 Variations in thermal behavior and moisture dynamics of crayfish subjected to dry-heat processing

#### 3.1.1 Core temperature profile and moisture content

**Fig. 1:**
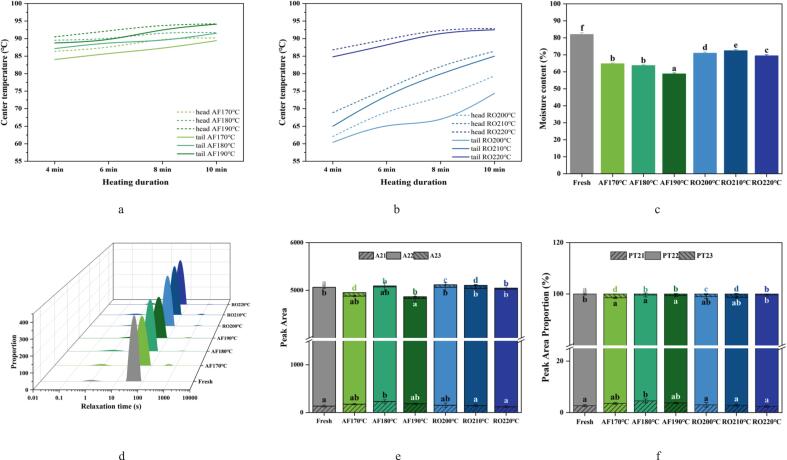

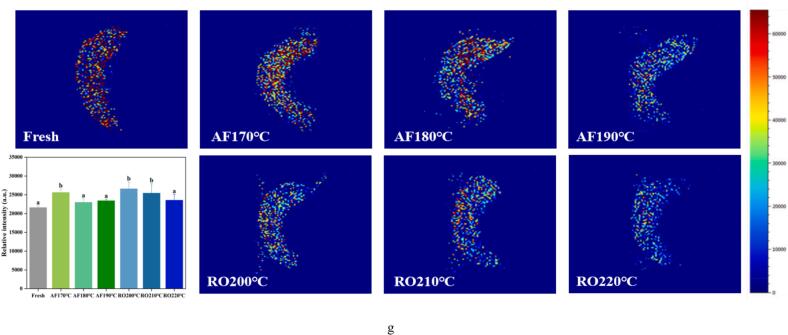
Thermal behavior and moisture dynamics in crayfish processed by air frying and roasting. (a) Core temperature profile during air frying, (b) Core temperature profile during roasting, (c) Moisture content comparison, (d) Transverse relaxation time (T_2_) distribution curves, (e) T_2_ peak areas, (f) Proportional distribution of T_2_ components, (g) Proton density maps with signal intensity histograms. The abbreviations “AF” and “RO” denote air frying and roasting groups, respectively. Different lowercase letters denote significant differences (*p* < 0.05) based on one-way ANOVA.

Fig. 1(a) and (b) depicted the temperature evolution within crayfish during 10-min dry-heat processing by air frying (AF) and roasting (RO), respectively. Distinct thermal dynamics were observed between anatomical regions. Initially, the cephalothorax temperature exceeded that of the abdomen, but this gradient diminished progressively with increasing heat penetration. Cumulative heat conduction and convective flux homogenization subsequently led to thermal equilibrium throughout the specimen. Higher processing temperatures significantly accelerated heat transfer kinetics, achieving dynamic thermal equilibrium. Core temperatures under both methods exceeded 75 °C, meeting food safety standards. AF demonstrated superior thermal efficiency, which is attributed to forced convection that rapidly distributes high-velocity heated air. In contrast, RO systems, reliant on radiative heating and natural convection, exhibited slower heat transfer rates (Wang et al., 2023), demonstrating the pivotal role of airflow dynamics in achieving uniform thermal processing. Fig. 1(c) showed significantly lower moisture content in AF-processed crayfish meat compared to RO (*p* < 0.05). This resulted from rapid surface moisture evaporation and crust formation induced by high-speed air circulation in AF. The significantly lower moisture content in AF samples (Fig. 1c) is a direct consequence of its superior convective heat transfer. High-velocity hot air rapidly evaporates surface moisture, creating a steep moisture gradient that drives intense internal water outward migration and crust formation. RO, with its dominant radiative heating, allows for a more gradual moisture redistribution, resulting in higher final moisture content despite similar core temperatures (Liang et al., 2022; Wang et al., 2023; Zhou et al., 2024).

#### 3.1.2 Water distribution

Water spatial arrangement in muscle tissues critically influences quality attributes, including edibility. Fig. 1(d) illustrated the CPMG transverse relaxation curve, depicting water dynamics in dry-heat processed crayfish. Fig. 1(e) and (f) summarized the peak areas and their ratios from low-field NMR measurements, clarifying water distribution changes with heating temperature and their impact on tissue quality. The inverse NMR T_2_ spectrum revealed three distinct peaks: T₂₁ (water tightly bound to macromolecules), T₂₂ (immobilized water within protein matrices), and T₂₃ (free water outside the myofibrillar network) (Li et al., 2021). Following dry-heat processing, the T₂₂ relaxation time decreased significantly from 47.69 ms to 38.72 ms. This reduction is consistent with findings in cooked pork (Cheng et al., 2019) and is primarily attributed to an enhanced chemical exchange between water and protein protons. This effect is driven by the denaturation, aggregation, and contraction of muscle fibers. The AF group exhibited lower T₂₂ values than the RO group, indicating stronger water-protein interactions under AF treatment. The T₂₂ component accounted for the majority of the signal proportion (>94 %), with minor contributions from T₂₁ (2–5 %) and T₂₃ (1–2 %). The decrease in A₂₂ and PT₂₂ values in the processed groups reflects a reduction in immobilized water content within the myofibrillar network. This loss is attributable to protein denaturation and contraction-induced water expulsion during heating. Compared to fresh samples, the immobilized water content (A₂₂) decreased in treated groups, while the free water content (A₂₃) increased. Furthermore, when comparing the two cooking methods, the AF group exhibited a higher proportion of bound water (PT₂₁) but lower proportions of immobilized water (PT₂₂) and free water (PT₂₃) than the RO group, respectively. This difference stems from distinct internal water redistribution patterns. The rapid dehydration in AF promotes the formation of a surface crust, which helps retain bound water internally. In contrast, the gradual moisture loss during RO facilitates a more extensive internal redistribution of water. This process results in higher proportions of immobilized and free water, likely due to the partial conversion of bound water (Cheng et al., 2019; Lian et al., 2023; Nikoo et al., 2014).

#### 3.1.3 Magnetic resonance imaging (MRI)

To elucidate dry-heat processing effects on crayfish moisture dynamics, MRI non-invasively visualized water distribution (Zhao et al., 2023). Fig. 1(g) displayed pseudo-color images of tail meat, where blue (low proton density) corresponds to free water and red (high proton density) to bound and immobilized water. The red areas diminished in both AF and RO groups compared to fresh samples and declined further with increasing temperature. This indicates progressive internal-to-surface moisture migration and drip loss during processing (Wang et al., 2021), consistent with moisture content trends (Fig. 1c). Mechanistically, high temperatures disrupt hydrogen bonds, converting bound water into free water. Initially, liberated protons aggregate locally via electrostatic attraction, elevating relative intensity. Subsequent temperature increases fully dissociate hydrogen bonds, randomizing proton distribution through thermal motion and reducing intensity (Lian et al., 2023). The results further imply that air-fried crayfish develops a crispy exterior with a tender interior, whereas oven-roasted crayfish tends to be juicy and plump.

### 3.2 Changes in protein oxidative degradation of crayfish subjected to dry-heat processing

#### 3.2.1 Protein oxidation

**Fig. 2:**
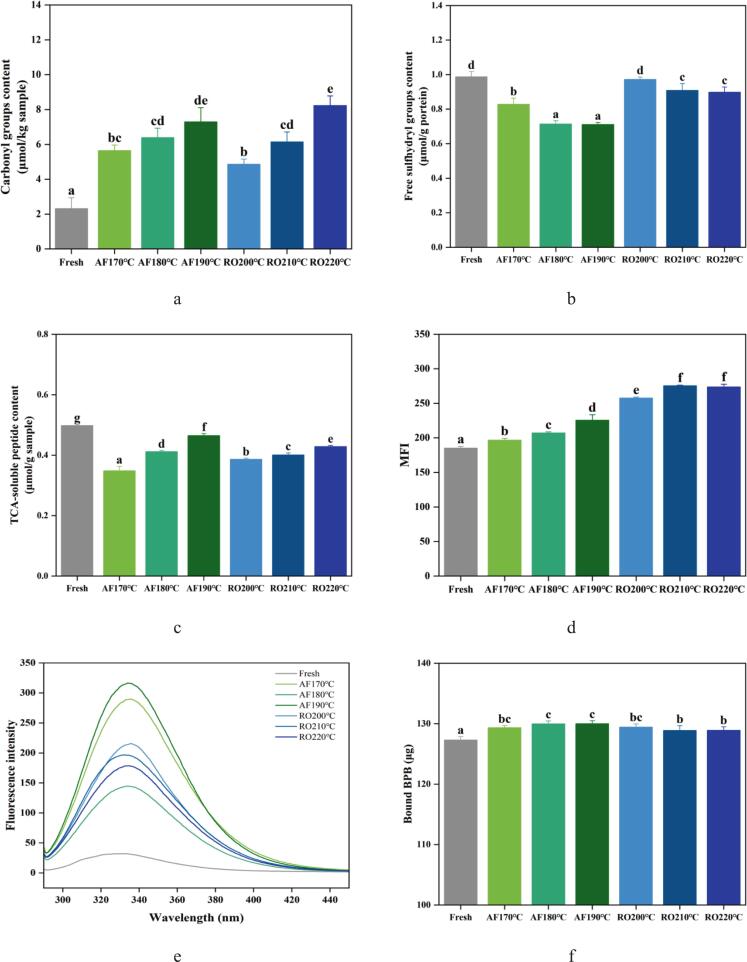

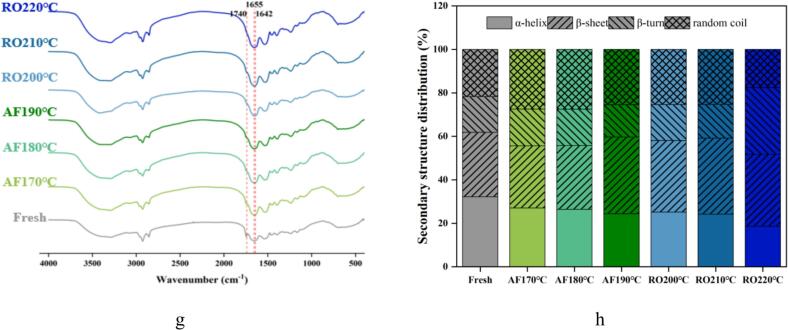
Protein oxidation and structural alterations in crayfish processed by air frying and roasting. (a) Carbonyl content, (b) Free sulfhydryl content, (c) TCA-soluble peptides, (d) Myofibril fragmentation index (MFI), (e) Intrinsic fluorescence spectra, (f) Surface hydrophobicity index, (g) FTIR spectra, (h) Secondary structure distribution. The abbreviations “AF” and “RO” denote air frying and roasting groups, respectively. Different lowercase letters denote significant differences (*p* < 0.05) based on one-way ANOVA.

Carbonyl content, a well-established indicator of protein oxidation (Shi et al., 2020), significantly increased (*p* < 0.05) in dry-heat processed crayfish versus fresh samples (Fig. 2a). This temperature-dependent increase was observed under both AF and RO conditions, with RO exhibiting a more pronounced elevation at higher temperatures. Maximum carbonyl content occurred in RO at 220 °C, indicating accelerated lysine residue oxidation and deamination forming carbonyl compounds (Wang et al., 2023).

Sulfhydryl groups (-SH), primarily located in the myosin head domain, are highly susceptible to oxidation by •OH, readily inducing intra- and intermolecular disulfide bonds (Huang et al., 2022; Zhu et al., 2022). Consequently, sulfhydryl reduction also characterizes protein oxidation. Both AF and RO significantly decreased free SH content versus fresh samples (*p* < 0.05, Fig. 2b). This oxidative modification displayed dose-dependency, as free SH declined progressively with increasing thermal exposure. This aligns with thermodynamic principles where elevated energy states promote intermolecular interactions, including disulfide cross-linking. The AF treatment resulted in a significantly lower free SH content (0.711–0.828 μmol/g protein) than RO (0.898–0.972 μmol/g protein). The more significant reduction in the content of free SH groups in AF samples suggests that AF might have facilitated a greater degree of sulfhydryl oxidation or intermolecular cross-linking. This is attributed to the synergistic effect of intense convective heating in AF and the consequent rapid dehydration. The forced convection not only delivers heat more efficiently but also quickly removes water, concentrating proteins and increasing the local probability of thiol group encounter and oxidation, thereby accelerating cross-linking compared to the gentler RO environment. Consistently, AF-processed scallop adductor muscle exhibited significantly lower residual free SH than roasted samples (Wang et al., 2023).

#### 3.2.2 Protein degradation

Trichloroacetic acid (TCA)-soluble peptides indicate protein hydrolysis extent (Li et al., 2022). Fig. 2(c) showed significantly lower TCA-soluble peptide content in dry-heat processed crayfish versus fresh samples (*p* < 0.05), attributable to thermal denaturation of endogenous proteases and reduced solubility of small peptides. Both AF and RO treatments exhibited temperature-dependent increases in peptide content. AF190°C yielded higher values (0.46 μmol/g) than RO220°C (0.43 μmol/g), suggesting prolonged thermal exposure facilitates peptide bond cleavage in myofibrillar proteins. Continuous high-temperature conditions likely generate additional low-molecular-weight fragments, enhancing TCA solubility. The short-duration, high-temperature exposure in air frying preferentially releases short peptides (Wang et al., 2025). Elevated TCA-soluble peptides correlate with improved sensory attributes, implying AF-processed crayfish may achieve superior crispness and flavor.

The myofibrillar fragmentation index (MFI) reflects myofibrillar protein hydrolysis and aggregation (Li et al., 2022). Fig. 2(d) demonstrated gradual MFI elevation in crayfish with increasing processing temperature. RO treatments exhibited significantly higher MFI than AF (*p* < 0.05), indicating more uniform myofibrillar breakdown under oven heating. This fragmentation pattern aligns with bound water loss dynamics (Fig. 1).

#### 3.2.3 Assessment of tertiary and secondary protein structures

Intrinsic fluorescence spectroscopy is a sensitive probe for tertiary structural changes in proteins, particularly regarding the microenvironment of tryptophan residues. The fluorescence spectra of crayfish proteins after dry-heat processing are shown in Fig. 2(e). Compared to fresh samples, heat treatment initially increased the fluorescence intensity. This rise is attributed to protein unfolding, which exposes previously buried tryptophan residues to a more hydrophilic environment and enhances their fluorescence (Wang et al., 2023). However, with further increases in processing temperature, the fluorescence intensity declined under both cooking methods. This subsequent decrease suggests the onset of fluorescence quenching, a phenomenon often associated with protein aggregation that can conceal aromatic residues (Li, Liu, et al., 2020). Interestingly, AF samples exhibited significantly higher fluorescence intensity than RO samples at comparable thermal doses, which indicates a distinct denaturation or aggregation pathway. While greater exposure of tryptophan due to oxidative denaturation can enhance fluorescence (Huang et al., 2022), the extent of quenching by aggregates is highly dependent on their morphology. The rapid dehydration and heating in AF may lead to the formation of a compact, cross-linked protein network. This structure could limit tryptophan mobility while maintaining many residues in a fluorescent state. In contrast, the slower RO process might facilitate the reorganization of proteins into larger, more condensed aggregates that effectively quench fluorescence. Therefore, the fluorescence signatures primarily reflect differences in the local chemical environment and the nanoscale architecture of protein aggregates, rather than indicating a straightforward correlation with the total amount of aggregation.

Surface hydrophobicity measured via bromophenol blue (BPB) binding reliably assesses protein denaturation. Thermal processing induces protein conformational shifts, exposing hydrophobic domains (Zhu et al., 2022). Fig. 2(f) showed enhanced surface hydrophobicity in AF and RO samples versus fresh samples. AF180°C and AF190°C exhibited significantly higher hydrophobicity than RO210°C and RO220°C (*p* < 0.05), indicating greater hydrophobic amino acid exposure for BPB binding in AF proteins (Wang et al., 2023). In RO crayfish, oxidative conditions enhance intermolecular interactions promoting polymerized assemblies. This aggregation likely masks unfolding effects or damages non-polar amino acids, reducing hydrophobic surface properties (Lian et al., 2023).

Fourier transform infrared spectroscopy (FTIR) analyzed dry-heat induced secondary structural changes in crayfish proteins (Fig. 2g and h). Amide *I* band frequencies (1700–1600 cm^−1^) quantified protein secondary components. Both air frying (AF) and roasting (RO) progressively decreased α-helix content while increasing β-sheet and random coil structures with rising temperature relative to fresh samples. This aligns with established thermal oxidation mechanisms where heat-induced protein oxidation reduces α-helices (1655 cm^−1^) and increases β-sheets (1642 cm^−1^) and random coils (Lian et al., 2023; Wang et al., 2023). A distinct carbonyl peak at 1740 cm^−1^ in processed samples corroborates elevated carbonyl content (Fig. 2a).

The stability of α-helices primarily depends on intramolecular hydrogen bonding between backbone -C=O and -N-H groups. Oxidative denaturation reduces these hydrogen bonds, manifested as diminished α-helix content and transition toward β-sheet conformation. Concurrently, dry-heat processing promotes protein aggregation through enhanced intermolecular hydrogen bonding, elevating β-sheet formation (Zhang et al., 2025). RO-treated samples exhibited lower cooking loss and higher protein mobility than AF counterparts. This increased kinetic accessibility strengthens intermolecular collisions, reinforcing hydrogen bonding networks and amplifying β-sheet formation (Wang et al., 2023). Air frying rapidly induced high-temperature dehydration, which synergistically drove protein oxidative cross-linking and conformational restructuring, consequently restricting the transformation of bound water to free water.

### 3.3 Modifications in the microstructure of crayfish muscle tissue after dry-heat processing

**Fig. 3:**
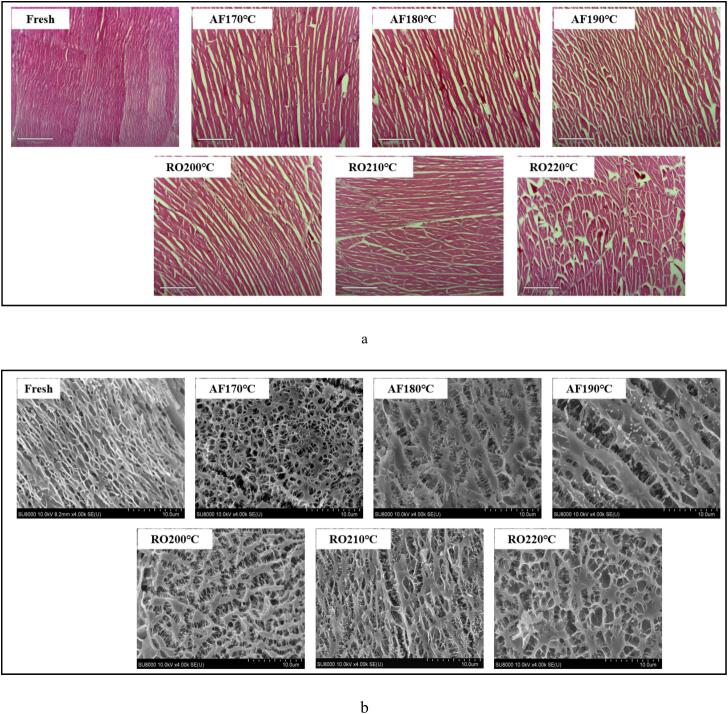
Microstructural alterations in crayfish muscle tissue processed by air frying and roasting. (a) Transverse section micrographs, (b) Ultrastructural features at 4000× magnification. The abbreviations “AF” and “RO” denote air frying and roasting groups, respectively.

Microstructural analysis (Fig. 3) revealed processing-dependent alterations in crayfish muscle fiber organization following dry-heat treatment. Such modifications reflect underlying protein structural changes in aquatic muscle foods (Li, Liu, et al., 2020). In fresh crayfish, H&*E*-stained cross-sections (Fig. 3a) showed well-defined, uniformly arranged muscle fiber bundles with small, dense pores. Dry-heat processing induced muscle fiber bundle aggregation and connective tissue separation. Increasing processing temperatures progressively enlarged intermuscular gaps, with distinct fiber fragmentation observed at peak temperatures (AF190°C, RO220°C). Air-fried samples developed compact muscle structures with limited fiber fragmentation due to rapid water loss inducing pronounced shrinkage and protein aggregation-driven matrix densification (Fig. 2). Conversely, roasted samples exhibited pronounced intermuscular gaps and progressive fiber disintegration. Complementing H&E observations, cold-field scanning electron microscopy (CF-SEM) at 4000× magnification revealed oxidative cross-linking transformed crayfish muscle proteins from ordered to disordered states (Fig. 3b). AF-treated samples displayed higher cross-linking density than RO counterparts, with void shrinkage proportional to temperature elevation (Fig. 1). This indicates rapid high-temperature processing induces protein network contraction and internal fiber rupture. These structural modifications critically influence water-holding capacity, which arises from capillary forces within myofilament networks, myofibrils, and extracellular spaces (Zhang et al., 2025). Myofibrillar proteins are critical for water binding and stabilization in these microstructural domains (Liang et al., 2022).

### 3.4 Alterations in sensory quality properties of crayfish subjected to dry-heat processing

#### 3.4.1 Color attributes

**Fig. 4:**
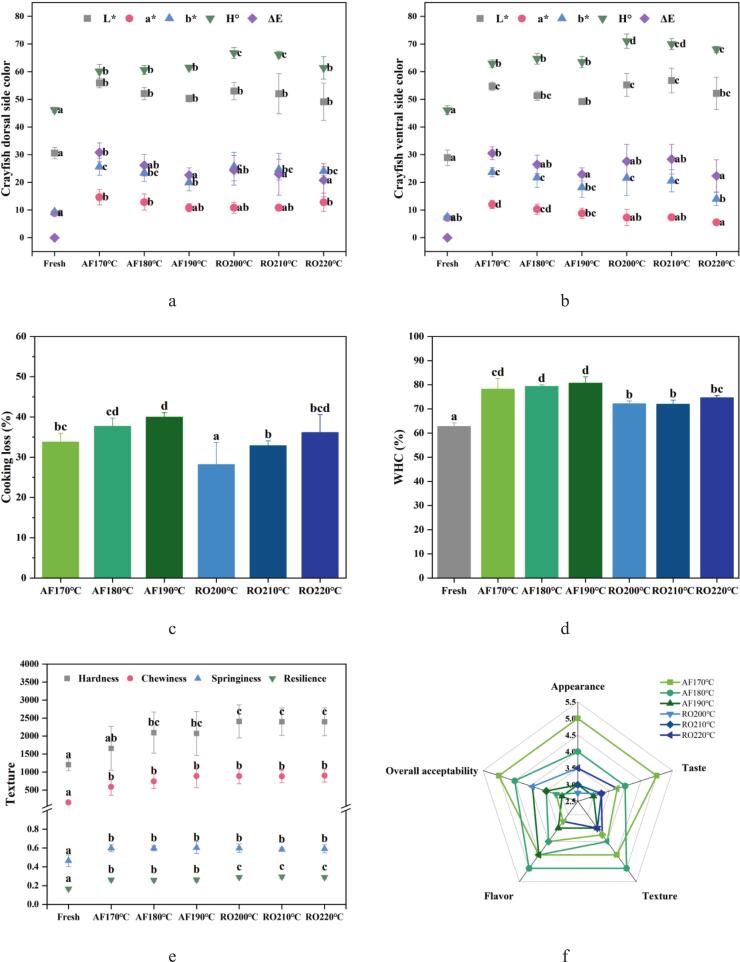

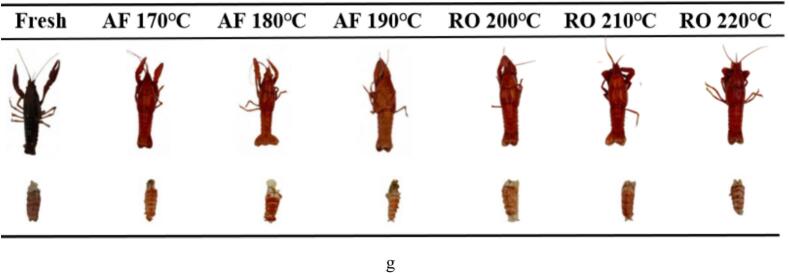
Quality attributes and sensory profiles of crayfish processed by air frying and roasting. (a) Dorsal surface color, (b) Ventral surface color, (c) Cooking loss, (d) Water holding capacity (WHC), (e) Texture profile analysis (hardness, chewiness, springiness, resilience), (f) Sensory evaluation radar chart (attributes: appearance, flavor, texture, taste, overall acceptance), (g) Visual appearance of cooked samples. The abbreviations “AF” and “RO” denote air frying and roasting groups, respectively. Different lowercase letters denote significant differences (*p* < 0.05) based on one-way ANOVA.

Surface coloration significantly influences consumer purchasing decisions and market value of crayfish, serving as the primary visual quality indicator. Fig. 4(a-b) present dorsal and ventral color parameters (L*, a*, b*, H^o^, ΔE) following dry-heat processing. Both surfaces exhibited consistent chroma trends: a* (redness) and b* (yellowness) decreased with prolonged heating. Initial a* elevation occurred through astaxanthin release from denatured myofibrillar proteins. Subsequent decline resulted from thermal degradation, structural isomerization, and oxidative decomposition of this pigment (Tsai et al., 2021). No significant color differences emerged between AF and RO groups. However, ΔE values (total color change versus raw samples) showed significantly higher alteration at AF170°C than AF190°C. This anomaly may be attributed to the forced convection and efficient hot-air circulation intrinsic to the design of the air fryer, where even minor temperature increments (20 °C) can induce pronounced shifts in ΔE. Hue angle (H^o^) analysis provided comprehensive color assessment: 0° (red), 90° (yellow), 180° (green), 270° (blue) (Li, Yuan, et al., 2020). RO samples exhibited significantly higher H^o^ values (67.25) than AF (*p* < 0.05), indicating AF induces more extreme color alterations under rapid high-temperature processing. Mechanistically, AF's intense Maillard reactions promote yellowing and darkening (Zhou et al., 2024), while RO yields homogeneous browning with lower chromatic variation. Pre-processing treatments may optimize AF coloration by regulating browning reactions to achieve desirable appearance (Zhou et al., 2024).

#### 3.4.2 Cooking loss and water holding capacity (WHC)

Fig. 4(c) demonstrated temperature-dependent increases in cooking loss for both air-fried (AF) and roasted (RO) crayfish, aligning with thermal processing trends reported for scallop adductor muscle (Wang et al., 2023). AF treatment yielded maximum cooking loss (39.97 %) at 190 °C, significantly higher (*p* < 0.05) than at 170 °C (33.79 %). Similarly, RO at 220 °C produced peak loss (36.13 %), significantly exceeding (*p* < 0.05) the 28.19 % observed at 200 °C. This phenomenon stems from This can be explained by the forced convection mechanism in AF: rapid surface moisture evaporation forms a dense crust that impedes internal water diffusion, while the accumulation of vapor pressure ruptures tissues (Fig. 1c and g).

Water-holding capacity (WHC) directly influences textural properties of processed crayfish. AF-treated samples exhibited significantly higher WHC than RO counterparts (*p* < 0.05, Fig. 4d), likely resulting from compact protein networks formed during rapid high-temperature processing. Despite lower overall moisture content, AF crayfish retain higher bound water proportions that enhance WHC. Conversely, RO's gradual heating produces looser protein structures where elevated free water content diminishes WHC, consistent with protein-water distribution mechanisms in crustaceans (Li et al., 2022). Collectively, these findings establish thermal intensity as the critical determinant of protein denaturation patterns and moisture retention efficacy in crustacean thermal processing.

#### 3.4.3 Textural properties

Texture analysis revealed distinct differences between the two processing methods. As shown in Fig. 4(e), the hardness of RO crayfish was significantly higher than that of AF samples. This textural divergence stems from their contrasting effects on protein structure. The rapid, high-intensity heating in air frying promoted extensive disulfide bond formation, as indicated by the pronounced reduction in free sulfhydryl groups (Fig. 2b). This process created a dense, cross-linked protein network which increased hardness and chewiness while helping retain a tender interior. Conversely, the gentler, more uniform radiative heat of oven roasting led to gradual dehydration and different structural changes. The higher myofibrillar fragmentation index (MFI) in RO samples (Fig. 2d) suggests more uniform protein breakdown. This resulted in a stiffer texture that was less cohesively cross-linked than in AF samples. These observations confirm that thermal-induced protein denaturation and cross-linking are primary modifiers of texture in muscle foods (Khalid et al., 2023; Li, Yuan, et al., 2020).

#### 3.4.4 Sensory evaluation

Fig. 4(f-g) presented appearance characteristics and sensory evaluation radar charts for air-fried (AF) and roasted (RO) crayfish. Dry-heat processing induced orange-red surface pigmentation and tissue contraction, resulting from astaxanthin liberation via Maillard reaction-mediated denaturation of carotenoid-protein complexes (Fig. 4a). AF produced more pronounced visual alterations than RO at identical durations, attributable to superior heat transfer efficiency through direct convective steam-laden air versus RO's radiative thermal transfer (Wang et al., 2023).

Sensory analysis of appearance, taste, flavor, texture, and overall acceptability demonstrated temperature-dependent divergence. AF samples achieved maximum total acceptability (24 points) at 170 °C, decreasing at higher temperatures despite peak aroma intensity at 180 °C from enhanced volatile compounds release. Conversely, RO exhibited ascending acceptability scores with temperature, culminating in optimal aroma at 220 °C. The superior texture profile of 170 °C AF samples, characterized by crisp exterior and tender interior, aligns with instrumental texture measurements (Fig. 4e).

### 3.5 In vitro digestive properties of crayfish protein subjected to dry-heat processing

#### 3.5.1 Ratio of insoluble residues

**Fig. 5:**
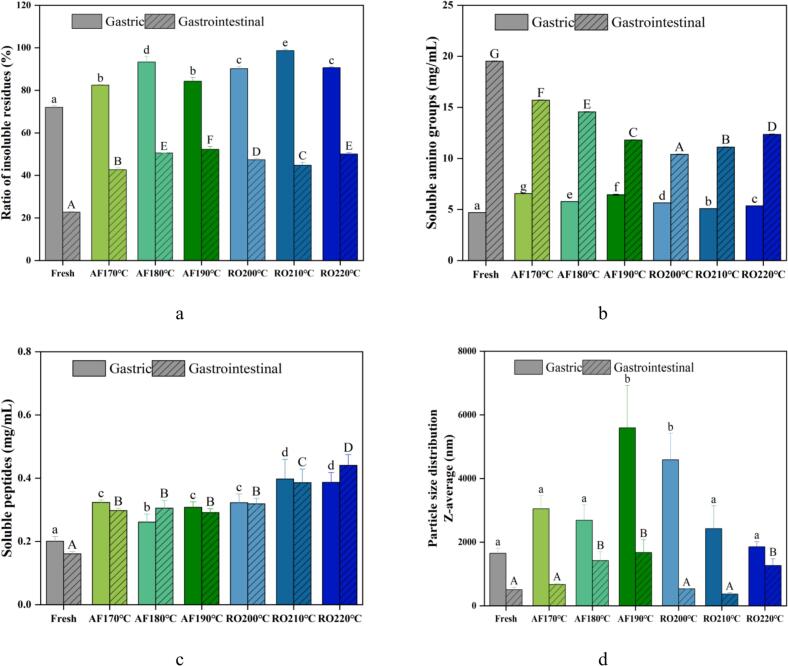
In vitro protein digestibility of crayfish processed by air frying and roasting. (a) Ratio of insoluble residues, (b) Free amino groups content (OPA method), (c) TCA-soluble peptides content, (d) Particle size distribution of digesta. The abbreviations “AF” and “RO” denote air frying and roasting groups, respectively. Different lowercase (gastric) or uppercase (gastrointestinal) letters indicate significant inter-stage differences (*p* < 0.05, one-way ANOVA).

The in vitro digestion characteristics of aquatic proteins depend on the extent of protein oxidation and degradation, structural alterations, and water distribution states. Heating intensity modulates protein spatial conformation, directly influencing insoluble residue formation during digestion. Dry-heated crayfish exhibited significantly higher insoluble residue proportions than fresh samples (*p* < 0.05, Fig. 5a), primarily due to heat-induced protein cross-linking and aggregation (Fig. 2). These structural modifications reduce proteolytic enzyme accessibility and mask essential enzymatic recognition sites (Hu et al., 2025). Insoluble residue ratios inversely reflect protein digestibility. Gastric-phase residues were substantially lower than gastrointestinal-phase values, as pepsin primarily generates short peptides with minimal amino acid release, while residual fragments undergo complete amino acid liberation via trypsin in intestinal digestion (Bai et al., 2023; Wan et al., 2024). After intestinal digestion, AF-treated samples showed marginally higher insoluble residue ratios (42.70 %–52.25 %) than RO-treated samples (44.80 %–50.05 %). This discrepancy likely stems from AF-induced moisture reduction (Fig. 1c), which diminishes protease-substrate contact efficiency.

#### 3.5.2 Soluble amino groups

Soluble amino group content serves as an indicator of enzymatic hydrolysis extent (Fig. 5b). Dry-heat processing significantly reduced soluble amino groups versus fresh crayfish (*p* < 0.05), indicating greater hydrolysis susceptibility in native protein conformations compared to heat-induced shrunken, cross-linked structures (Fig. 2). Post-gastric digestion revealed temperature-dependent dynamics: AF170°C yielded peak values (7.18 mg/mL), significantly exceeding AF180°C (6.32 mg/mL) and AF190°C (7.05 mg/mL). This inverse correlation with insoluble residues (Fig. 5a) demonstrates temperature-sensitive proteolysis where moderate heating (170 °C) enhances protein solubility and enzyme accessibility, while higher temperatures promote denaturation/cross-linking that impedes digestion. Intestinal-phase digestion progressively increased soluble amino groups. The AF170°C sample exhibited a 1.4-fold increase (17.18 mg/mL) versus gastric phase, whereas the 190 °C sample showed only modest elevation (12.92 mg/mL, 0.8-fold). This trend aligns with the patterns of intestinal insoluble residues, confirming that high-temperature-induced protein cross-linking obstructs pepsin degradation and subsequently suppresses the efficacy of pancreatic enzymes (Bhat et al., 2021).

#### 3.5.3 Soluble peptide content

Notably, RO-treated samples exhibited temperature-dependent increases in soluble amino acid content (11.38, 12.15, 13.52 mg/mL at ascending temperatures). Oven processing induced protein structural relaxation, exposing additional enzymatic cleavage sites that enhanced digestibility and promoted peptide and amino acid release. This mechanism is substantiated by soluble peptide measurements, which serve as multifunctional indicators reflecting both digestive characteristics and nutritional properties. Fig. 5(c) demonstrated significantly higher soluble peptide content in RO samples versus both AF and fresh counterparts (*p* < 0.05), confirming superior digestive performance in oven-roasted crayfish compared to air-fried preparations.

#### 3.5.4 Particle size distribution

During gastrointestinal digestion, crayfish proteins undergo enzymatic degradation by pepsin and trypsin, converting high-molecular-weight proteins into smaller peptides or amino acids (Wang et al., 2025). This proteolysis reduces digest particle size. Fig. 5(d) presented particle size distributions following dry-heat processing and digestion. Fresh crayfish exhibited minimal gastric-phase particle size, indicating efficient initial pepsin-mediated proteolysis. In AF samples, gastric particle size increased progressively with temperature (170 °C: 3047.33 nm, 190 °C: 5593.00 nm), correlating with insoluble residue proportions (Fig. 5a). Although intestinal digestion reduced particle sizes across treatments, AF190°C retained significantly larger particles than AF170°C. This persistence demonstrates high-temperature-induced protease-resistant structures limit complete degradation, restricting macromolecular conversion even after intestinal processing (Zhao et al., 2019). Such size differentials critically influence enzyme-substrate contact area and nutrient absorption efficiency. Contrastingly, RO samples exhibited smaller particle sizes at 210–220 °C than at 200 °C (4587.33 nm). This observation aligns with the increased protein degradation (higher MFI) observed under these conditions (Fig. 2d), which likely contributed to a finer initial breakdown. Notably, particle size increased again after intestinal digestion (531.00–1268.00 nm), suggesting the formation of stable aggregates that resist pancreatic enzymes (Wan et al., 2024).

Collectively, the in vitro digestion data reveal a consistent mechanism driven by protein network density. Air frying formed a tightly cross-linked, low-moisture protein matrix, evidenced by reduced T₂₂ relaxation times and increased surface hydrophobicity (Figs. 1d and 2f). This compact structure impeded protease access, resulting in higher insoluble residues, larger particle sizes, and lower peptide release. In contrast, oven roasting promoted a more open and uniformly fragmented protein network, reflected by a higher myofibrillar fragmentation index (Fig. 2d). This structural openness facilitated enzymatic penetration and proteolysis, ultimately explaining the smaller digestive particle sizes and superior digestibility of RO samples.

### 3.6 Shifts in volatile compounds of crayfish subjected to dry-heat processing

**Fig. 6:**
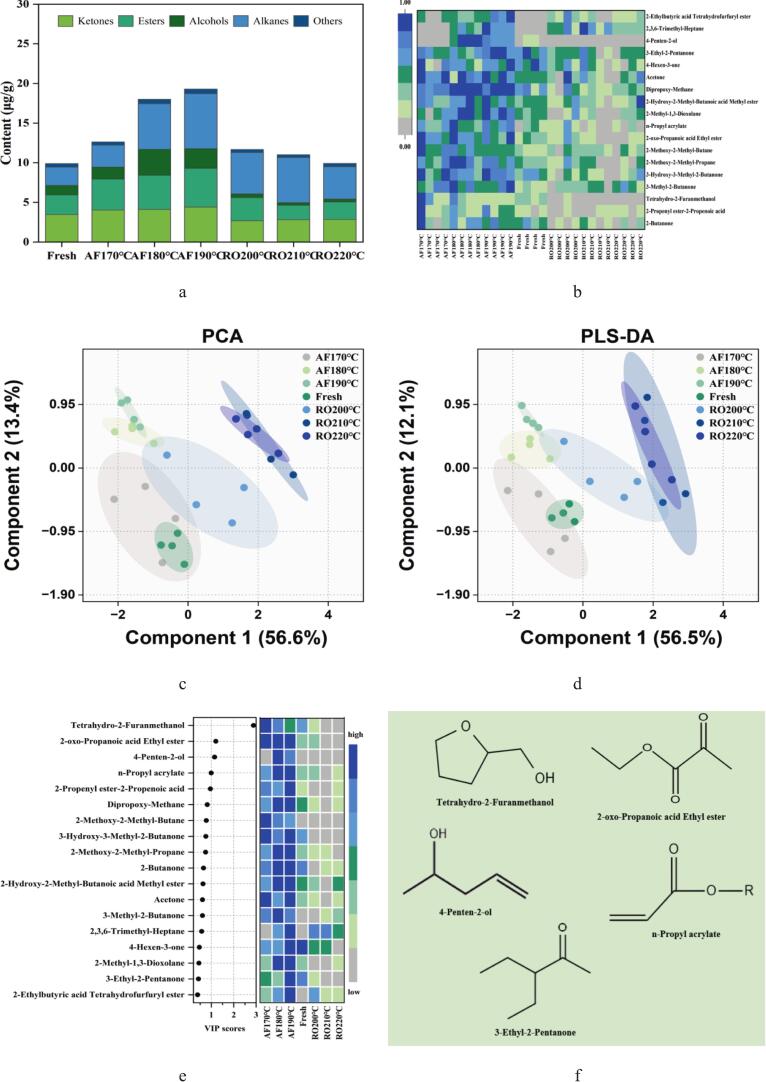
Volatile compound profiling of crayfish processed by air frying and roasting. (a) Volatile categories distribution, (b) Heatmap of volatiles, (c) Principal component analysis (PCA) score plot, (d) Partial least-squares discriminant analysis (PLS-DA) score plot, (e) Variable importance in projection (VIP) scores of volatiles, (f) Chemical structure of signature compounds. The abbreviations “AF” and “RO” denote air frying and roasting groups, respectively.

The characteristic flavor of cooked crayfish originates from complex interactions among flavor precursors, intermediates, and reaction products (Sohail et al., 2022). Dry-heat processing generates volatile compounds through lipid oxidation, Maillard reactions, condensation and esterification pathways. These reactions collectively produce alcohols, ketones, esters, and other volatile compounds that define the sensory profile. Fig. 6(a) categorized volatile compounds identified across treatment groups, including 6 ketones, 5 esters, 2 alcohols, 4 alkanes, and 1 other compound. Comprehensive volatile compounds evaluation employed thermal mapping, principal component analysis (PCA), partial least-squares discriminant analysis (PLS-DA), and variable importance projection (VIP) scoring (Figs. 6b-e), identifying five key compounds: tetrahydro-2-furanmethanol, ethyl 2-oxopropanoate, 4-penten-2-ol, n-propyl acrylate, and 3-ethyl-2-pentanone (Fig. 6f).

Significant compositional and proportional differences in volatile flavor compounds emerged between AF and RO crayfish. AF samples contained higher volatile compounds concentrations (12.63–19.30 μg/g), increasing with temperature elevation. Conversely, RO samples showed lower levels (9.91–11.71 μg/g) that decreased at higher temperatures. This divergence indicates AF's rapid high-temperature processing promotes volatile compounds generation through thermal protein aggregation (Shen et al., 2020). Conversely, RO samples showed lower overall volatile compounds levels, which further decreased at higher temperatures. This trend contrasts with AF, where rapid surface dehydration likely forms a crust that helps sequester volatile compounds. In RO samples, the slower and more uniform heating may not create such a restrictive barrier. Instead, it could favor the continuous evaporation and loss of volatile compounds throughout the extended cooking process. Furthermore, the more porous protein matrix observed in RO samples (Fig. 3) might offer less physical entrapment for flavor compounds, facilitating their diffusion and loss rather than retention. Zhang et al. (2023) demonstrated reduced moisture with extended heating (e.g., microwaving) enhances desirable aromas in tilapia. Processing conditions critically determine protein structures that govern flavor retention (Jiang et al., 2022), while compound volatility depends on molecular properties and mass transfer resistance (Bai et al., 2023). Structural conformation and surface hydrophobicity alterations during processing further modulate flavor accumulation and release (Shen et al., 2020).

Cluster heatmap analysis (Fig. 6b) visualized treatment-specific volatile profiles, revealing reduced abundance in RO versus Fresh and AF groups. AF samples exhibited prominent accumulation of characteristic compounds 2-butanone and tetrahydro-2-furanmethanol. Ketone formation arises through multiple biochemical pathways, primarily involving thermal oxidation of unsaturated fatty acids and amino acids, β-scission events during lipid peroxidation, keto-enol tautomeric equilibria, and secondary oxidation processes affecting hydrocarbon substrates (Luo et al., 2022). Short-chain ketones like 2-butanone impart buttery notes, with concentration dynamics significantly correlating with thermal parameters and potentially modulating aroma through volatility thresholds (Luo et al., 2022). Furan derivatives, essential flavor carriers formed during thermal processing, arise primarily through carbohydrate thermal decomposition, along with Maillard reaction-mediated condensation and oxidative pathways involving polyunsaturated fatty acids. AF's intense convective heating likely accelerated these pathways, yielding higher furan levels than RO. The elevated levels of furan derivatives in AF samples are consistent with findings in fried surimi (Luo et al., 2022), suggesting that rapid, high-temperature processing is a common driver for such reactions across aquatic protein systems. However, our study further identifies tetrahydro-2-furanmethanol as a marker specific to the air-frying process of crayfish, a distinction not reported in earlier studies on fish or surimi. This could be due to the unique combination of sugars and amino acids in crayfish, or the particular moisture removal kinetics of air frying. PCA (Fig. 6c) explained 70 % of the total variance (PC1:56.6 %, PC2:13.4 %), demonstrating clear intergroup discrimination. PLS-DA modeling (Fig. 6d) revealed finer separation, RO clustered in quadrants I and IV, while Fresh and AF groups occupied II and III with temperature-dependent spatial displacement, suggesting synergistic aroma modulation mechanisms. VIP scoring identified tetrahydro-2-furanmethanol (VIP > 2.5) as the optimal discriminator (Fig. 6e). This oxygen heterocycle likely derives from catalytic hydrogenation of Maillard intermediate furfural. Notably, AF processing enhanced Maillard reactivity, as evidenced by the temperature-dependent accumulation of tetrahydro-2-furanmethanol, establishing this compound as a process-specific marker. Key discriminatory compounds also included ethyl pyruvate, 4-penten-2-ol, propyl acrylate, and 3-ethyl-2-pentanone (Fig. 6f).

### 3.7 Correlation analysis of physicochemical indicators and edible quality

**Fig. 7:**
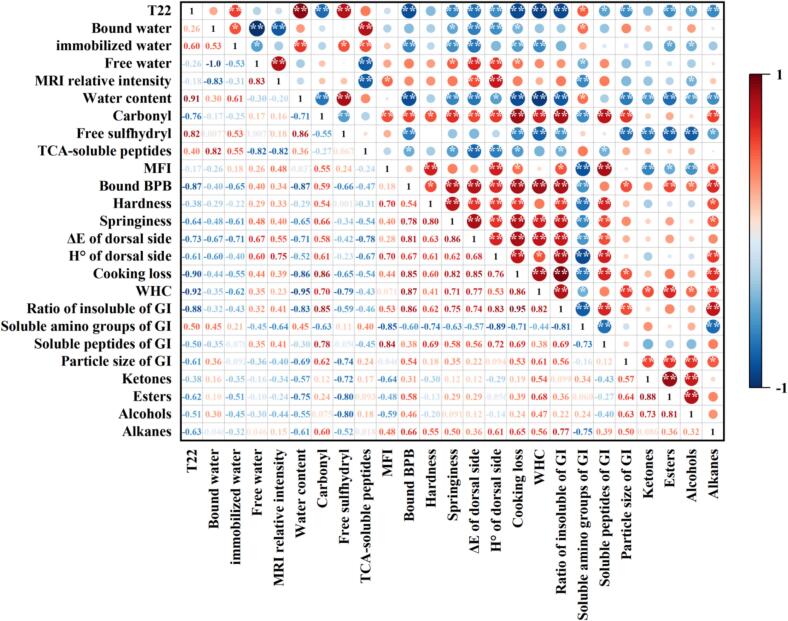
Correlation matrix of physicochemical attributes and edible quality in processed crayfish. Color intensity reflects association strength (red: positive; blue: negative), with asterisks denoting significance levels (*p < 0.05, **p < 0.01). (For interpretation of the references to color in this figure legend, the reader is referred to the web version of this article.)

Pearson correlation analysis was used to evaluate the correlations between dry-heat processing parameters and the edible quality (texture, color, flavor, digestibility) of crayfish in relation to water migration dynamics and protein structural modifications. Fig. 7 visually depicted positive (red) and negative (blue) correlations through color-coded representation. This study revealed significant negative correlations between moisture content, bound water and sensory attributes (hardness, springiness, color attributes, WHC, cooking loss), whereas free water positively correlated with these attributes. These relationships indicate that bound water retention results from protein aggregation-induced densification of crayfish muscle tissue during dry-heat processing. In contrast, free water maintains tissue turgor, delays protein aggregation, and functions as the primary sensory quality regulator. Protein carbonyls, MFI, and surface hydrophobicity demonstrated positive correlations with adverse sensory properties. Conversely, free sulfhydryl groups and TCA-soluble peptide content exhibited negative correlations. In terms of digestive characteristics, carbonyls and surface hydrophobicity positively associated with insoluble residues in gastrointestinal digestion. While carbonyls and MFI correlated positively with soluble peptides in digesta, they showed negative correlations with free amino groups. These patterns collectively demonstrate that densely cross-linked protein networks suppress digestibility. The interplay between protein structural changes (initiated by oxidative degradation) and moisture dynamics is crucial in modulating the digestibility of crayfish proteins. Thermal processing readily induces protein oxidation, which modifies amino acid side chains and reduces enzymatic susceptibility, consequently impairing digestive properties (Wang et al., 2023). Simultaneously, limited free water availability hinders protease-substrate interactions, while protein aggregation sterically masks proteolytic cleavage sites, further reducing digestion efficiency (Zhao et al., 2019). Volatile compounds (ketones, alcohols, esters, alkanes) positively correlated with water holding capacity, surface hydrophobicity, and digesta particle size, but negatively with free sulfhydryl groups, MFI, and moisture content. This suggests hydrophobic flavor compounds co-precipitate with protein aggregates, enhancing flavor retention while compromising bioaccessibility (Wan et al., 2024). These correlation patterns fundamentally underscore the trade-off inherent in dry-heat method selection. AF's rapid, high-intensity process strongly correlates with indicators of protein aggregation and flavor development but also with reduced digestibility. RO's gentler approach shows the opposite associations, prioritizing structural integrity for digestion over intense flavor and texture formation.

## 4 Conclusions

This study demonstrates that air frying and oven roasting distinctly regulate crayfish quality through divergent water-protein dynamics. Air frying accelerates dehydration and protein oxidation, fostering a crispy texture and enriching flavor compounds such as tetrahydro-2-furanmethanol. In contrast, oven roasting promotes more uniform water distribution and proteolytic degradation, resulting in a porous structure that enhances digestibility but at the expense of flavor intensity. These contrasting outcomes reveal an inherent trade-off between sensory appeal and digestibility governed by the processing method. The elucidated mechanisms, which specifically address how convective intensity and heating rate dictate water migration and protein aggregation pathways, provide a clear rationale for future process optimization. For instance, moderating initial dehydration in air frying or adjusting heating phases in roasting could be promising strategies to better balance texture, flavor, and nutritional quality. Overall, this work establishes water-protein-thermal interactions as fundamental determinants of multi-scale quality in crustacean processing.

## CRediT authorship contribution statement

**Wensi Xu:** Writing – original draft, Investigation, Formal analysis, Data curation. **Shengcai Xu:** Software, Methodology, Data curation. **Aihua Deng:** Supervision, Software, Project administration. **Xiaoyang Liu:** Resources, Funding acquisition. **Liang Song:** Validation, Supervision. **Dayong Zhou:** Supervision, Project administration. **Qifu Yang:** Writing – review & editing, Visualization, Funding acquisition.

## Declaration of competing interest

The authors declare that they have no known competing financial interests or personal relationships that could have appeared to influence the work reported in this paper.

## Data Availability

Data will be made available on request.
